# The Metric System in Prescriptions

**Published:** 1877-01

**Authors:** 


THE METRIC SYSTEM IN PRESCRIPTIONS.—

*To the Editor of the Western Lancet:*

SIR—At the present time the metric system is used by scientific men, physicians included, nearly all over the civilized world, be it to express capacity, length, or weight. In the United States it has also been legalized for some time past. French and German medical authors use it to the exclusion of every other system; so that, to read their works and comprehend them, it becomes necessary to be conversant with it. It does not complicate matters, but simplifies them, as will be seen from what follows. We now use ounces of three different weights, to-wit: ℥ j. (avoirdupois), = 437.5 grains, fl. ℥ j. = 455.55 grains, and ℥ j. (Troy), = 480 grains. Nor was this all. Before the almost universal adoption of this system, every country had its own medical weight or weights, each one differing from the other. It is not necessary to give illustrations of this, for everybody knows it. In view of these facts, it is evident that it will do away with numerous totally different weights (as it has done wherever it has been adopted), and will substitute ONE, which will be easily deciphered, and represent the same quantity everywhere, making it possible for a prescription to be understood and correctly compounded by any competent apothecary in any part of the world. It cannot be questioned but that it is a step in the right direction toward securing an universal medical "language," the desirability of which was so ably set forth by Dr. Edward Seguin, before the Centennial Medical Congress at Philadelphia, just closed. Its acquisition is not beset with insurmountable obstacles—quite the contrary, it is very easy. One need not be gifted with mathematical talents of the highest order to learn it. Any person of average ability ought to be able to master the art of using the metric system, in writing prescriptions, within a very short space of time. It requires no more ability to do this than we have a right to expect every one to possess to whose care the reputations, the health, aye, the lives, of his fellowcreatures are intrusted. All that is required is to remember that a gramme is equivalent to about fifteen grains, and that thirty grammes correspond to about an ounce (Troy); that is all. In prescribing by the metric system, the quantity of each ingredient is expressed in multiples or fractions (decimal) of a common unit, which in this instance will be the grammes, thus:

| | Grammes. |
|---|---|
| R.—Pulv. Opii, . . . . . . . | 0.2. |
| Plumbi Acetatis, . : . . . | 2 0. |
| M. Ft. pil. No. xij. | |

Or,

| | Grammes. |
|---|---|
| R.—Pulv. Gallae, . . . . . . | 4.0 |
| Ungt. Aq. Rosae, . . . . . | 30.0 |
| M. Fiat ungentium. | |

A little practice will enable the prescriber to THINK in it, and save him, subsequently, the trouble of translating his ideas into it. Further, I venture to express the opinion here that he who has used it will be so pleased with it that he will never travel the old road. If all this be so, how does it happen that it is not more extensively employed in this country? Simply because the majority of us will not take the trouble to learn it, being satisfied with things as they are. Let those at least adopt it who are not too old to learn new things, and who are not too strongly wedded to old ways. May they who desire the profession in this country to keep pace with it in others,

"Ring out the old, ring in the new;
Ring out the false, ring in the true."

Should this rapidly-written communication be the means of causing those who are not familiar with the metric system of weights and measures to examine into its merits, it will have attained its object, as it will most certainly then gain many strenuous advocates for its general use—a thing which will, I think, occur within this decade.

Yours truly,

ALFRED EMANUEL REGENSBURGER.

*San Francisco, November* 2, 1876.

